# Immunological Features in the Process of Blood Platelet-Induced Alloimmunisation, with a Focus on Platelet Component Transfusion

**DOI:** 10.3390/diseases7010007

**Published:** 2019-01-14

**Authors:** Olivier Garraud, Fabrice Cognasse, Pierre Moncharmont

**Affiliations:** 1 EA_3064, Faculty of Medicine of Saint-Etienne, University of Lyon, 42023 Saint-Etienne, France; fabrice.cognasse@univ-st-etienne.fr; 2 Institut National de la Transfusion Sanguine, 75015 Paris, France; 3 Établissement Français du Sang Auvergne-Rhône-Alpes, 69150 Décines, France; pierre.moncharmont@efs.sante.fr

**Keywords:** transfusion, platelets, platelet components: haemovigilance, alloimmunisation

## Abstract

Alloimmunisation to platelet antigens is not uncommon; a large number of females, having had pregnancies, developed antibodies to Human Leukocyte Antigen (HLA) moieties harboured on their foetuses’ cells (inherited from the father(s)) that may conflict with further pregnancies and transfused Platelet Components occasionally. This is possible since platelets constitutionally express HLA class I molecules (though in copy numbers that consistently differ among individuals). Platelets also express HPA moieties that are variants of naturally expressed adhesion and aggregation molecules; HPA differences between mothers and foetuses and between donors and recipients explain alloimmune conflicts and consequences. Lastly, platelets express ABO blood group antigens, which are rarely immunising, however transfusion mismatches in ABO groups seem to be related to immunisation in other blood and tissue groups. Transfusion also brings residual leukocytes that may also immunise through their copious copy numbers of HLA class I (rarely class II on activated T lymphocytes, B cells, and dendritic cells). In addition, residual red blood cells in platelet concentrates may induce anti-red blood cell allo-antibodies. This short review aims to present the main mechanisms that are commonly reported in alloimmunisation. It also critically endeavours to examine paths to either dampen alloimmunisation occurrences or to prevent them.

## 1. Circumstances of Patient Immunisation

Foreign platelets can be exposed to the body and the immune system in two circumstances:

(i) Exposure most commonly occurs during pregnancy where micro-haemorrhage can occur at different times during pregnancy but most often at the onset of placental circulation and during the perinatal period. New-born platelets are half-foreign but can expose antigenic molecules made up after encoding by genes inherited by the father. None of the salient platelet antigens encoding genes are carried by sexual chromosomes and reciprocally all genes are transmitted in an autosomal (co)dominant manner. The mother’s immune system may develop active and productive immunisation against antigens unknown by the mother and corresponding to the father’s. Human platelet antigens—referred to as HPA [1]—are not strictly speaking exclusive to platelets as some can be found on other cells, but they usually characterise platelets [2]. Foetus to mother immunisation then leads to mother to foetus passive transfer of antibodies that destroy platelets displaying cognate antigens, exposing the foetus and the neonate to thrombocytopenia and bleeding. The most severe consequence is usually intracerebral haemorrhage and subsequent severe disability [3]. Foetal neonatal alloimmune thrombocytopenia (FNAIT) has been covered by several recent reviews. All antigens seem to not be associated to similar severity and novel—rare—HPA antigens are still being discovered or remain to be identified [4,5,6,7,8,9]. Of note, there has been recent evidence experimentally claiming that intracranial haemorrhage occurs after immunisation against antigens exposed on the ß3 (GPIIIa, which harbours numerous variants of the highly prevalent (in Caucasians) HPA1a) chain of platelet adhesion molecules and not the ß1. The ß3 chain has a role in angiogenesis and impairment results in haemorrhage; intravenous immunoglobulin injection can reverse this devastating occurrence [10]. Special interest is now given to one such molecule harbouring HPA specificities, namely GPIbα (CD42b), a molecule that matters as it protects platelets from degrading upon stresses (a property that can have importance in transfusion processes) [11,12,13]. Alloantibodies targeting GPIbα may lead to Fc-independent thrombocytopenia, which is resistant to injectable immunoglobulin treatment [14]. Occasionally, immunisation occurs against non-HPA antigens.

Most cases of pregnancy-associated immunisation, however, occur in the Human Leukocyte Antigen (HLA) system, and mainly relate to HLA class I moieties (though anti-HLA class II antibodies (Abs) can be detected). Depending on the surveys and techniques used to measure Abs, it is estimated that ~17% of females exhibit anti-HLA Abs after one pregnancy, with increases after each pregnancy [15,16].

(ii) The second occurrence of immunisation with platelets is subsequent to transfusion, as suggested very soon after transfusion became available [17], and as deciphered by Dutcher in 1981 [18]. It is essentially seen after platelet component (PC) transfusion. Technically, platelet antigen immunisation is possible after transfusion of whole blood, though this type of component is infrequent in economically developed countries. Red blood cell component transfusion can immunise against HLA moieties as a consequence of residual leukocytes that express high levels of HLA class I molecule, also present on platelets. Leukoreduced BCs have been shown to immunise considerably less than non-leukoreduced controls towards HLA class I, as illustrated by Andreu et al. (1988; [14]) initially, and then by the TRAP study (2005; [19,20,21]). Both Andreu [22,23] and Slichter [24] provided evidence that UV-irradiation partly inactivates leukocytes and helps limit alloimmunisation. However, stringently leukoreduced BCs (to less than the 10^6^/BC standard) can still immunise. Multiparous females, who develop anti-HLA class I antibodies after PC transfusion three times more than nulliparous females, also have an increased risk of posttransfusion immunisation [20]. Patients receiving platelet component transfusion usually endure repeated transfusion of platelet components and often of packed red blood cell concentrates as well, as they tend to be pan-cytopenic in general. Of important note, they may also be transfused with therapeutic plasma if exposed to severe bleeding. Therapeutic plasma is a blood product rich in polyreactive immunoglobulins that may interfere with the immunisation outcome by dampening the reaction. Patients immunised by platelets manifest with three types of immunisation, even all three or targeted to one only. Firstly, patients can be immunised by platelet antigens that are HPAs as seen above. Then, they can be immunised by HLA class I variants (platelets express this set of molecules, however, in numbers that vary greatly among individuals alongside ABH antigens [25] and always in a smaller number of copies than on leukocytes) [26]. Lastly, they can be immunised by residual red blood cells and antigenic moieties, as minimal amounts of them may be highly immunogenic (RhD for example). In addition to these circumstances, some patients may be reactive to platelet (HLA I) antigens as they have been immunised by cell or organ transplantation.

**Figure 1:**
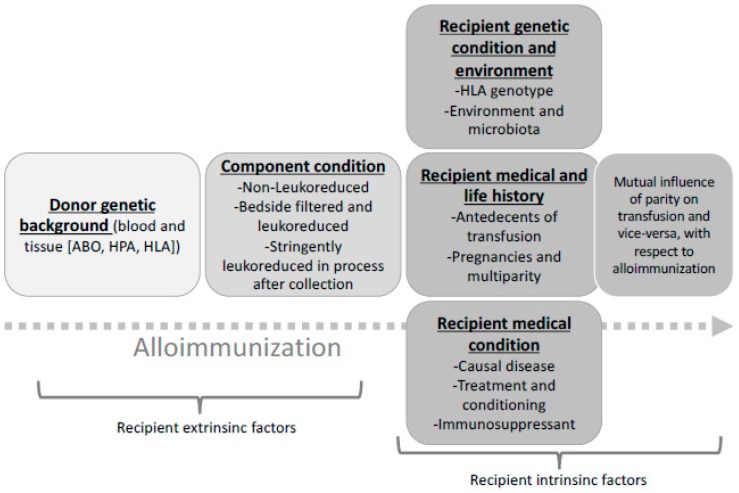
Major conditions pertaining to alloimmunisation against antigens carried by allogenous blood cells. This cartoon depicts, from left to right, extrinsic (donor- and donated blood component-linked) parameters and intrinsic (own recipient-linked) factors.

As can be seen, immunisation with platelet and red blood cell derived immunogenic moieties is complex as it results from numerous events linked to the transfused cells and also to the recipient’s genetics, disease, and treatment (and especially immunosuppressant regiments or interfering drugs). Other variables have recently been sought such as environment and microbiota. Figure 1 summarises and illustrates this complex situation. In all, there might be both intrinsic and extrinsic parameters pertaining to alloimmunisation that cannot be properly addressed in human pathology but modelled in mouse systems.

## 2. Allogenous (Foreign) Blood Cell Related Parameters Involved in Recipient Immunisation

**Figure 2:**
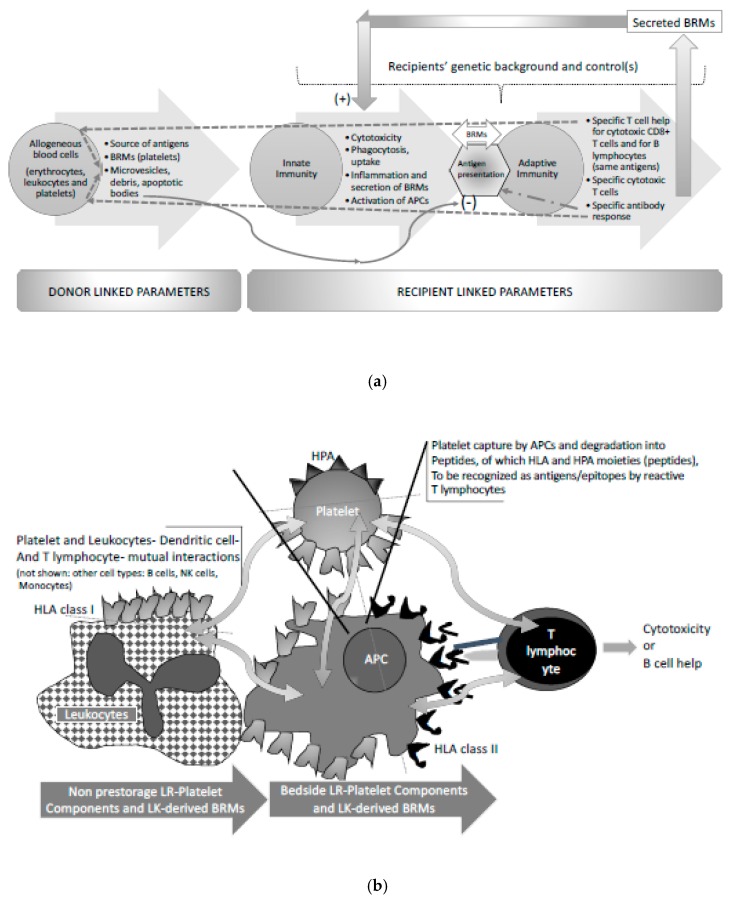
T-lymphocyte dependency of adaptive immune responses to foreign blood cell antigens harboured by allogenous blood cells. (**a**) This cartoon synthesizes the immunological steps from the incoming of sources of antigens to the different possible outcomes. BRMs stand for Biological Response Modifiers; APCs stand for Antigen Presenting Cells. (**b**) This cartoon illustrates (synthesizes) the mutual relationships between on the one hand platelets, and on the other hand leukocytes, dendritic cells, T-lymphocytes (shown), as well as other cell types such as B lymphocytes. Of particular notice, platelets and leukocytes in non-leukoreduced (LR) platelet components activate each other and secrete huge amounts of Biological Response Modifiers (BRMs) that vary in composition upon the storage time. It is hypothesized that this type of secretion alters the dendritic cell (and other Antigen Presenting Cell (APC) functioning) and influences alloimmunisation. The cartoon next introduces the main steps of antigen recognition, likely favoured by the cytokine and chemokine rich milieu.

Some patients, especially those suffering from malignancies, become chronically thrombocytopenic due to central and/or peripheral causes. In turn, such patients may become platelet transfusion-dependent. Repeated exposure to foreign platelets exposes to immunisation [27]. The pathophysiology of platelet antigen immunisation can be modelled in a classical sequence of a T-cell-dependent response as illustrated in Figure 2a,b. Immunisation is quite a common feature when platelet components include leukocytes, for several reasons that are illustrated in Figure 2. Consistent leukoreduction has significantly reduced but not abrogated the occurrence of this complication, as in certain patients few residual leukocytes seem to immunise well against HLA antigens.

The main feature of platelet immunisation is clinical, with a two-step process in general: (i) transfusion inefficacy, to below the expected yield, and then (ii) transfusion refractoriness. An estimated 30% of immunised patients become refractory to PC transfusion [28]. Another pathology, that is fortunately a rare event, is post-transfusion purpura (which will not be further discussed in this mini-review) [29]. Transfusion inefficacy is defined by a platelet count that remains low after transfusion of a platelet component, confirmed by a Current Count Increment (CCI) below 7. The CCI is calculated as follows: (CCI = (PLT count after transfusion − PLT count before transfusion) × Body surface area (m^2^) × 100: number of transfused PTLs (×10^11^)), “PLT” standing for “platelets”. Refractoriness is suspected when ABO compatible or—preferably—ABO identical platelets, no more than 72 h old and adjusted to the dose according to the ad hoc formula, fail to restore the platelet count, with CCI still below 7 [30].

Transfusion inefficacy/refractoriness is in fact complex. An elegant review by Pavenski et al. (2012) reports on causes that are mainly ascribed to as non-immune (~80%) such as fever, bleeding, disseminated intravascular coagulation, sequestration [26], etc. Reciprocally, ~20% are caused by antibodies: isolated anti-HLA (~80%), isolated anti-HPA (~15%) and combined anti-HLA + HPA (~5%). Of important note, autoantibodies can make pathologic determination more complex. As can be seen from the above, Abs are central to the definition of immune refractoriness to platelet transfusion. Bearing in mind that Ab detection tests mainly detect plasma-free, cell unbound, anti-HLA/HPA Abs, there is much room for undetected, platelet bound-Abs, making the 20%/80% immune versus non-immune causalities questionable. Elution tests would probably help clarify the figures.

Abs to HLA moieties can be lymphocytotoxic or non-lymphocytotoxic, as determined in batteries of tests. However, the Luminex^®^ technology tends to substitute cellular assays. The nature and occurrences of anti-HLA, HPA and CD36 antibodies have been described in a recent journal article [31]. Next, several mechanisms have been deciphered that pertain to alloimmunisation Ab-induced thrombocytopenia: platelets opsonised by IgMs and IgG1-3 bind FcγRIII^+^ monocytes/macrophages and are phagocytosed [32,33]. Certain IgG may signal and activate platelets through FcγRIIa molecules intra or extracellularly. This mechanism seems to depend on Abs to certain epitopes and also would reveal FcγRIIa variants [34,35]. Abs to GP1ba can activate platelets to produce sialidases which alter the glycocalyx and expose Galactose residues. Such platelets are phagocytosed by certain hepatocytes [36].

## 3. Fine-Tuned Mechanisms of Alloimmunisation: As Yet Unexplored

**Figure 3:**
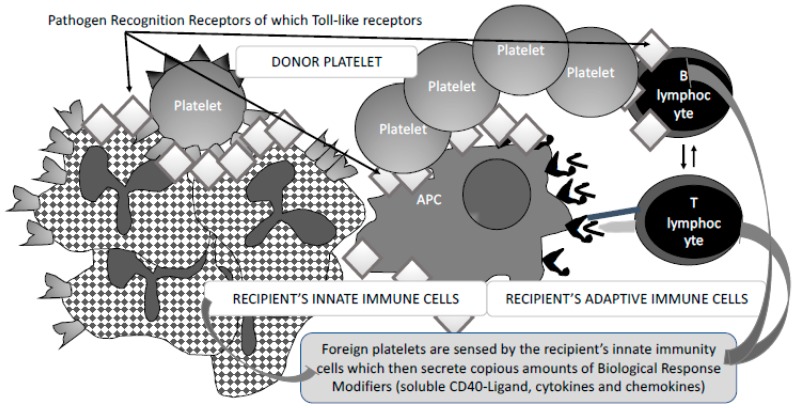
The making of a sustained adaptive immune response to allogenous blood cell antigens is largely supported by the created pro-inflammatory environment. This figure illustrates the likely importance of innate immunity involvement that ends up with a potentially strong pro-inflammatory response, especially in the case of platelets that have the potential of sourcing the immune system environment with molecules that are essential to antigen presentation and adaptive immunity.

**Figure 4:**
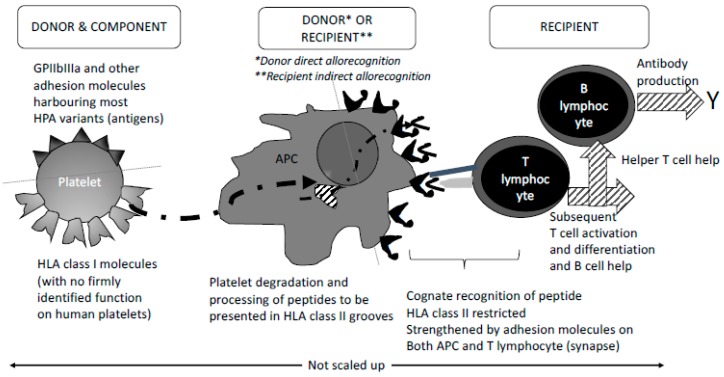
A proposed sum up of the mechanisms pertaining alloimmunisation to foreign platelet antigens. From donor (**left**) to component (**centre**) and to recipient (**right**), this cartoon aims at summarising events that explain the process of pathogen capture, antigen presentation, and cognate recognition, followed by a potentially robust antibody production, with clinical consequences.

The fine-tuned mechanisms of productive immunisation remain quite an unsolved mystery for a large part, as a high number of PC-transfused patients and multiparous persons remain non-immunised. Immunosuppression due to either the causal disease or the drug regimen cannot by itself explain that certain patients under an intensive immunosuppression regimen can develop productive immunisation with high titre antibodies. As illustrated in Figure 3, there may be several lines of explanation. Foreign platelets (red blood cells, leukocytes) present with a variety of major and minor differences, some sensed as antigenic variants and some as pathogen associated molecular patterns or PAMPs. PAMP sensing—signalling non-self-associated biological danger—initiates membrane and intracellular activation of phagocytes and dendritic cells, and memory B-cells if previous sensitisation has occurred. This initiates the pro-inflammatory component of innate immunity that is essential for antigen presentation to reactive T lymphocytes. Of note, platelets that can be activated through the process of leukocyte contact in danger sensing are prone to secrete large amounts of pro-inflammatory molecules collectively termed Biological Response Modifiers (BRMs) [37,38]. Several of these factors and some undiscovered factors can interfere with all the cell partners of the innate inflammatory response and antigen presentation, and of the adaptive response (Figure 4). One such BRM copiously produced by activated platelets is CD40L, which, in its soluble form (sCD40L) and/or its membrane bound form, can bind Antigen Presenting Cell (APC) CD40 and B cell CD40. Such binding may activate APCs and differentiate B cells, as seen in experimental models [39,40,41]. As an example, cocultured platelets and B lymphocytes were mutually activated (increased expression of platelet CD62p and B-cell CD86). Platelet/B-cell interactions were characterised in particular by changes in membrane expression of CD40 and CD40L by both platelets and B lymphocytes. Moreover, there was a significant, platelet-dependent reduction of sCD40L and RANTES mRNA expression. After 3day’s incubation with platelets, differentiated B cells increased their in vitro production of IgG1, IgG2, and IgG3, but not IgG4, IgA, or IgM [42]. Moreover, B cell recall response is probably eased because of co-activation through Pathogen Recognition Receptors (PRRs) and B cell receptor for antigen. Antigen binding through antibodies may strengthen the reactive B cell through additional FcγRs and Complement Receptors. Antigen presentation is essential and central as it instruments the presentation to reactive T-cells to become helper effectors and sustain reactive B cell differentiation. Alloimmunisation stands for a typical T-cell-dependent mechanism, likely enhanced by BRMs that prevent reactive cells entering apoptosis and favour differentiation (there again, activated platelets may play a role through the panoply of molecules secreted, including sCD40L). Nevertheless, the constraint of antigen presentation is the capacity of professional presenters to bind antigenic-derived material. Antigen presentation thus appears central. It has been shown experimentally that allo-recognition can result from either of two mechanisms: (i) direct recognition by recipient T-cell receptor of HLA-derived peptides presented by donor APCs, a mechanism which has become unlikely at a time where stringent leukoreduction has almost eliminated foreign APCs; (ii) donor HLA-derived soluble peptides bind to recipient APCs [26,43]. Experiments aimed at blocking APC and reactive T-cell contact by cross linking CTLA4-Ig indeed prevented immunisation [44,45,46]. As has been proposed for red blood cell immunisation in the mouse system [47], one cannot rule out that expansion of T_H17_ T cell clones, or T_FH_ clones that are exclusive to secondary follicles in lymph nodes and strongly supportive of B cell maturation into Ab producing cells electively dampen T_REG_ clones, the only cells supporting tolerance. On the other hand, there was experimental evidence that allogenous platelets can further induce some level of immunosuppression in eliminating CD8+ T cell clones and, in turn, increasing skin graft survival in mice. Further, in a recent and very elegant study, Saris et al. evidenced that platelets endocytosed by dendritic cells—through likely apoptotic mechanisms (apoptotic platelets)—have the potential of eliciting strong IFN-γ production by alloreactive CD4+ T-cells. This further suggests that not only HLA from residual leukocytes but also HLA class I from foreign platelets themselves can be immunising [48]. This tends to demonstrate that the outcome is complex and multifocal. Next, extensive studies on FNAIT have revealed that some mothers displaying certain HLA genotypes (such as DRB4*01:01 and foremost DRB3*01:01 [49,50,51,52]) presented with HPA1-derived peptides more efficiently than their negative counterparts. Similar findings have been obtained in red blood cell immunisation [53] and transplanted organ immunisation where certain HLA genotypes (and minor histocompatibility antigens MICA and MICB) associate with strong donor-specific antibodies or DSAs [54]. Not all alloimmunisation susceptibilities through HLA genotypes have been deciphered as yet. Meanwhile, it is remarkable that some platelet component recipients develop Abs to a fast-increasing array of HLA and often HPA antigens following each transfusion episode, with dozens of reactive alloantibodies, rendering transfusion extremely unproductive (those super-presenter/responder individuals, often females having been already sensitised to HLA moieties through pregnancies, may develop auto-antibodies as well). Lastly, it has been observed that ABO-incompatible platelet components might favour immunisation to platelet antigens additive to possible anti-A or -B isoagglutinins [55].

## 4. Implications for Patient Management

Patient management involves two mainstreams: upstream prevention at the blood establishment level and downstream at the hospital blood bank level.

The prevention phase itself involves two directions. One was set up two decades ago in many places and consists in leukodepleting (leukoreducing) blood soon after donation, before storage (referred to as “prestorage”) to levels below 10^6^ residual leukocytes per blood component. This practice has been assessed in many surveys so far and it was concluded that this is a residual level where antigen load may not be sufficient in the majority of patients to be productively immunising [56]. Also, this step significantly reduces the production of proinflammatory BRMs released by leukocytes and especially neutrophils [57,58]. Prestorage leukoreduction is much more effective than bedside leukofiltration to reduce immunisation, inflammation, and a side effect, which we recently evidenced, that is the activation of platelets in platelet components. Indeed, in this study, platelets in platelet-rich plasma constituting one unit were induced to secrete copious amounts of inflammatory BRMs, favouring a vicious circle for immunisation [59]. The second direction is still observational. More and more blood establishments undertake implementation of pathogen-inactivation or reduction technologies for platelet components. Industrial partners tend to claim that such processes are believed to help mitigate antigen presentation and immunisation as a secondary outcome [60]. Despite encouraging results observed in animal models [61], a recent observational study disproved this [62] and raised the alerted to the issue that at least one such process might on the contrary favour alloimmunisation when platelet components are aged, calling for precaution [63]. 

Where platelet components are leukoreduced during prestorage and can be selected in an ad hoc inventory, the downstream step consists of specifically selecting or choosing the component. This aims to maximise the expected outcome (platelet count recovery) and minimise the harmful side effects. The standard approach is a dual approach, as it targets both quantity and quality in terms of platelet components: (i) The quantity option, in a severely allo-immunised bleeding patient or in a patient exposed to a high risk of bleeding, consists of opting in favour of a high-dose of platelets or continuous infusion of low doses of platelets to overcome antibodies by competition mechanisms [64,65,66,67]. Concomitantly or alternatively, infusion of intravenous (polyreactive) immunoglobulins can be offered to compete with pathogenic antibodies and to target B lymphocytes through ITIM signalling caused by crosslinking of Igs on FcγRIIb receptors. Therapeutic plasma exchange and replacement with fresh frozen plasma can be a last resort in emergency situations [68]. In the longer term, drug regimens can be sought, such as anti-CD20 therapy and corticosteroids, to down regulate production of pathogenic (mainly anti-HLA) allo-antibodies and autoantibodies. (ii) The qualitative option consists of matching homologous platelet components. Single-donor apheresis platelets can be HLA and HPA typed in regular donors to build up a database and support computer-assisted delivery, which is a good option when the patient does not have alloantibodies directed at too many HLA/HPA antigens. This policy could prevent alloimmunisation of the recipient and/or make cross-matching between donor Ags and patient Abs easier. As for organ transplantation, authorised and forbidden donor HLA antigens are defined. Lastly, HLA matchmaker assistance can be an option in well-equipped pathology departments [68,69,70,71,72]. One such strategy has been presented in a recent review of ours [31]. Platelet cross-matching is nevertheless the main possibility in the event of severe alloimmunisation. Desensitisation by low dose, continuous platelet transfusion has been proposed [73]. Strategies aiming at managing platelet refractoriness are, however, deductive, as has been consistently outlined in several review articles [74,75,76,77]; there are too few randomized controlled trials to validate strong recommendations. Nevertheless, both blood services and scientific societies made recommendations based on Grade C evidence; they all specify that HLA-matched transfusions usually benefit the patient. These data are stronger on the short term (1 h CCI) than on the longer term (24 h CCI). To overcome this caveat, it is the more and more preferred to use Eplet matched-HLA antigen pairing than epitope pairing [65,66,67].

Hospital blood banks and reference HLA and platelet immunology laboratories often opt for combinations of all strategic lines, which are presented to the prescribing clinician for a concerted decision. The check-point for each arm of the transfusion strategy is the presence of or the high risk of bleeding. An attentive attitude may otherwise be an option.

## 5. Concluding Remarks

Immunisation due to platelet antigens (foetus to mother) and platelet components (transfusion) is not uncommon [78,79,80,81,82,83,84] and may lead to clinical situations that are difficult to manage, at different levels: prevention of bleeding, transfusion strategy, decision-making as to suppressant prescription. Prevention methods would be better amplified by all means mitigating transfusion-related alloimmunisation to platelet moieties. One evidence-based measure is ensuring ABO compatibility or identity anytime this is achievable [85,86]. Along the same lines, RhD alloimmunisation of Rhesus-negative platelet component recipients should be prevented by RhD matching where possible, and/or prevention with anti-D serum prophylaxis if available. This strategy is often reviewed nationwide according to recommendations [87]. Another measure is not yet convincingly evidence-based but highly possible (randomised clinical trials are indeed difficult to promote and survey in this matter), and that is the freshness of platelet components (less than or more than three days after collection/processing, as extrapolated from general considerations on platelet component transfusion-associated hazards) [38,40,41,88]. More studies seem, however, to be required to confirm this strategy, even in retrospective cases. Use of platelet inactivation or reduction technologies may not be the appropriate answer to this problem, which is somehow a disappointment. It is, however, to be followed up on a larger scale, as data would be available. Strategies aimed at strengthening computer databases of volunteer platelet donors accepting extensive genotyping, and strategies consisting of genotyping at-risk recipients would be worth assessing for feasibility, costs, and benefits in an overall strategy in the health care setting and in blood establishments.
